# Detection of Atmospherically Relevant Mixed Mercuric Compounds by Chemical Ionization Mass Spectrometry

**DOI:** 10.1021/acsearthspacechem.6c00061

**Published:** 2026-05-19

**Authors:** Mohammad Borna Bahramsari, Duyen B. Nguyen, Na Mao, Joel Duzha, Michael S. Eberhart, Alexei F. Khalizov

**Affiliations:** † Department of Chemistry and Environmental Science, 5965 New Jersey Institute of Technology, 161 Warren Street, Newark, New Jersey 07102, United States; ‡ Department of Chemical and Materials Engineering, New Jersey Institute of Technology, 161 Warren Street, Newark, New Jersey 07102, United States

**Keywords:** chemical ionization mass spectrometry, mixed mercuric compounds, gaseous oxidized mercury, Raman spectroscopy

## Abstract

A significant fraction of atmospheric mercury enters the aquatic and terrestrial environments via the deposition of gaseous oxidized mercury (GOM). The photochemically produced GOM comprises many molecules, but most experimental work employs HgCl_2_ and HgBr_2_ as surrogates. This calls for a more realistic representation of GOM for the development of detection and sampling methods. Here, several mixed mercuric compounds XHgY (X and Y are Cl^–^, Br^–^, I^–^, NO_2_^–^, and NO_3_^–^) were synthesized by exchange reactions involving mercuric and nonmercuric compounds, isolated in crystalline form, and characterized by Raman spectroscopy. Vapor above these solids was analyzed by ion drift–chemical ionization mass spectrometry (ID-CIMS), using the SF_6_^–^, HNO_3_·NO_3_^–^, and I^–^ reagent ions. When X and Y are both halogens, XHgY readily volatilizes and can be detected by ID-CIMS, based on unique ion products XHgY·F^–^, XHgY·NO_3_^–^, and XHgY·I^–^. On the contrary, mixed mercuric salts containing at least one nonhalide ligand cannot be volatilized and detected. Furthermore, solid ClHgNO_2_ and BrHgNO_2_ rapidly oxidize upon exposure to ambient air, and the oxidation is accompanied by hydrolysis to form red HgO. We conclude that ClHgNO_2_ and BrHgNO_2_ are insufficiently redox-stable to survive multiday preconcentration from ambient air on sorbents and insufficiently volatile to be thermally desorbed for analysis in their original form.

## Graphical abstract

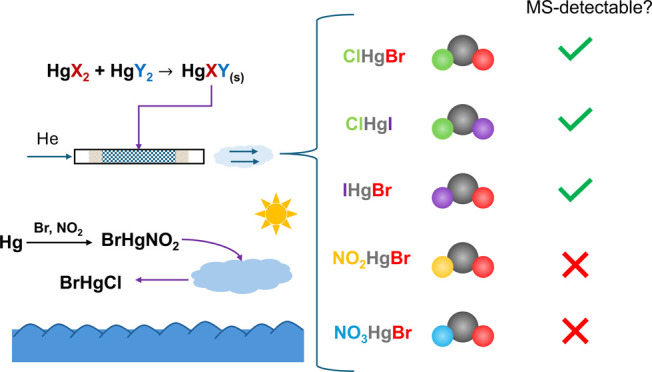

## 1 Introduction

Mercury is a highly toxic pollutant entering the environment from both geogenic and anthropogenic emission sources.^1,2^ The atmosphere is an important intermediate compartment for mercury, with the dominant form being gaseous elemental mercury (GEM). The atmospheric lifetime of GEM, six months to a year, is long enough for it to be transported on hemispherical and even global scales in the air.^3,4^ The long-range atmospheric transport results in global impacts of mercury, contrary to other heavy metals that typically disperse within a locale or region. Environmental impacts of GEM are exacerbated when it is converted to gaseous oxidized mercury (GOM), which can be deposited rapidly to aquatic and terrestrial ecosystems, methylated, and biomagnified in biota, especially in the food chain where human exposure occurs.^5^ Once inside the body, the methylmercury can cross the blood–brain barrier and damage nervous, digestive, and immune systems even after a limited exposure.^6,7^ The ability to predict mercury cycling in the environment and its impacts is hindered by an insufficient understanding of atmospheric mercury chemistry, including reaction mechanisms and molecular speciation of GOM.^8–10^ This has led to many studies focused on developing various techniques for quantification and identification of GOM in field and laboratory settings.^11,12^

A major challenge in detecting atmospheric GOM is its ultratrace concentration (at tens of parts per quadrillion by volume, corresponding to tens pg m^–3^),^13^ requiring ultrahigh detection sensitivity. Many existing methods rely on sample preconcentration, followed by desorption, thermolysis, and analysis as elemental mercury.^14–17^ Direct operational speciation into GEM and GOM has been achieved in dual channel methods, using atomic fluorescence to measure Hg^0^ corresponding to total and oxidized mercury.^12,18,19^ However, direct molecular speciation of atmospheric GOM presents a significant challenge. Although a very recent study^20^ has shown the in situ online detection of individual mercuric halides in the polar boundary layer by an atmospheric pressure chemical ionization mass spectrometer (APCI-MS) specifically designed for ultratrace atmospheric measurements,^21^ implementing this direct method in polluted urban environments may be far from straightforward, necessitating preconcentration on sorbents. To obtain molecular speciation of GOM, reversibly binding adsorbing media can be used in conjunction with temperature-programmed thermal desorption. In the most robust implementation, which has been employed in the field, GOM desorbing during a temperature ramp is pyrolyzed and analyzed as GEM.^17,22,23^ Furthermore, there have been attempts to replace the pyrolysis stage with chemical analysis of the evolving GOM by mass spectrometry.^24,25^ In Jones et al.^24^ thermally desorbed GOM was separated in a gas chromatograph equipped with a cryogenic preconcentrator and then analyzed with an electron impact ionization mass spectrometer (GC–MS), whereas Deeds et al.^25^ analyzed the thermally desorbed GOM using a standard laboratory APCI-MS. In these studies, only the relatively volatile HgCl_2_ and HgBr_2_ were successfully detected, but the collection-desorption-analysis approach failed for Hg­(NO_3_)_2_ and HgO. Only by using the direct injection probe could Hg­(NO_3_)_2_ be detected,^24^ indicating that this chemical either has too low a vapor pressure or is too sticky to pass through the GC column and associated plumbing. The direct probe analysis failed to show any molecular ions for HgO,^24^ in agreement with quantum chemical calculations, predicting that HgO is weakly bound in monomeric form.^26,27^

Another major analytical challenge is related to the labile nature of Hg­(II) compounds, which readily exchange ligands upon alterations of the surrounding chemical environment. This exchange can occur not only in solutions,^28^ but also on surfaces,^24,29^ potentially leading to speciation artifacts. Indeed, the formation of ClHgBr has been reported in the GC-MS-based system used for preconcentration and detection of HgCl_2_ and HgBr_2_.^24^ Our recent study^29^ has confirmed the exchange between two mercuric chemicals, HgCl_2_ and HgBr_2_, to form ClHgBr on both inert and active surfaces, as well as shown the occurrence of a rapid exchange upon the interaction of Hg­(II) with nonmercuric chemicals. For instance, aqueous Hg­(NO_3_)_2_ in the presence of excess Cl^–^ was converted to HgCl_2_, which was detected in solution as HgCl_3_^–^ and in headspace above solid as HgCl_2_F^–^, using electrospray ionization mass spectrometry (ESI-MS) and SF_6_^–^ CIMS, respectively.^29^ If such exchange reactions occur on atmospheric aerosols, the original photochemically generated GOM species (e.g., BrHgOH, Hg­(OH)_2_, BrHgNO_2,_ and HOHgOOH, as suggested by quantum-chemical studies^30,31^ ) would be converted to other forms of Hg­(II), among which the most volatile would repartition back to the gas phase. Indeed, recent global modeling studies of the mechanism of atmospheric mercury chemistry using photostable HgCl_2_ to represent the new species formed through aerosol processing, show significant revolatilization of GOM taken by sea-salt aerosols.^32,33^ Halogen ions are enriched in aerosols relative to seawater^34^ and also often show an additional enrichment at the surface of sea-salt aerosol droplets,^35–37^ potentially promoting the formation and subsequent volatilization of various mixed mercuric species XHgY, such as HgCl_2_, ClHgBr, and possibly ClHgNO_3_.

When both X and Y are halogens, the existence of XHgY in the gas phase has been spectroscopically demonstrated.^38^ Their crystallographic structures have been elucidated using X-ray diffraction,^39–41^ and their nature in both solid state and solution has been investigated by Raman spectroscopy.^42^ Furthermore, these compounds appear to form during the analysis of surface-preconcentrated mercuric halides.^24,25,29^ The experimental evidence becomes ambiguous when either X or Y is not a halogen. While it has been reported that permeation tubes loaded with Hg­(NO_3_)_2_ and HgO emit chemicals that behave like GOM, e.g., can be collected by Hg­(II) traps, such as nylon membranes and KCl-coated denuders,^17^ their chemical nature is unknown. BrHgNO_2_ and BrHgNO_3_, suggested by quantum-chemical studies as possible candidates for atmospheric GOM in the polluted boundary layer, are stable in the gas phase but photolabile.^30,31^ BrHgNO_2_ is produced through a two-step pathway involving the reaction of bromine radicals with GEM to form BrHg·, followed by reaction of BrHg· with NO_2_.^43^ In contrast, BrHgNO_3_ forms in a three-step process, whereby BrHg· reacts with O_3_ to produce BrHgO·, followed by reaction of BrHgO· with NO_2_.^33^ However, these compounds have not yet been synthesized in the laboratory, and it is unclear whether they are vaporizable or stable in the condensed phase.

Accordingly, our goal was to explore the detection of mixed mercuric chemicals XHgY (with X and Y being Cl^–^, Br^–^, I^–^, NO_2_^–^, or NO_3_^–^) in the gas phase upon thermal desorption of their solid samples. We synthesized several types of XHgY in an aqueous solution, using different approaches, isolated the reaction products, identified some of them by Raman spectroscopy, and explored the detection of their vapors by ion drift–chemical ionization mass spectrometry (ID-CIMS), using SF_6_^–^, HNO_3_·NO_3_^–^, and I^–^ as reagent ions. Additionally, we employed the Average Dipole Orientation (ADO) theory in conjunction with quantum chemical calculations to determine the rate constants of reactions between some of the XHgY molecules with the three reagent ions for estimating the theoretical sensitivity under our detection conditions.

## 2 Methodology

### 2.1 Chemicals and Gases

Mercuric chloride (HgCl_2_, Honeywell, >99.5%), mercuric bromide (HgBr_2_, Alfa Aesar, >99%), mercuric iodide (HgI_2_, Sigma-Aldrich, >99%), mercuric nitrate monohydrate (Hg­(NO_3_)_2_·H_2_O, Strem Chemicals, >98%), and silver nitrite (AgNO_2_, Strem Chemicals, >98%) were used for the synthesis of mixed mercuric compounds. Helium and nitrogen compressed gases of ultrahigh purity (UHP), supplied by Airgas, were used in ID-CIMS. Sulfur hexafluoride (SF_6_, Airgas 15% in argon), nitric acid (HNO_3_, Avantor, 70%), and methyl iodide (CH_3_I, Alfa Aesar, >99.5%) were used as precursors of reagent ions.

### 2.2 Synthesis of Mixed Mercuric Compounds

XHgY compounds, where X and Y are both halogens, can be readily synthesized by exchange reactions involving individual monohalides HgX_2_ and HgY_2_ ^44^

1 $${HgX}_{2}+{HgY}_{2}⇌2XHgY$$

Table 1 shows that equilibrium constants are sufficiently large to ensure a significant conversion using an equimolar ratio of reactants. For instance, starting with aqueous solutions of 0.01 M HgCl_2_ and 0.01 M HgBr_2_, one may expect a 3.7-fold excess of the reaction product ClHgBr over each reactant at equilibrium. Since room temperature (r.t.) solubility of mercuric halides decreases significantly in the series HgCl_2_, HgBr_2_, HgI_2_ (0.269, 0.0171, and 1.3 × 10^–4^ M, respectively^45^), using equimolar solutions for synthesis was not always feasible. In the case of HgCl_2_ and HgBr_2_, an acceptable amount of ClHgBr could be produced by mixing equal volumes of their solutions saturated at r.t., corresponding to a 16:1 HgCl_2_ to HgBr_2_ molar ratio. The product mixture obtained under such conditions contained a negligible amount of HgBr_2_, but a significant excess of HgCl_2_, estimated as 0.01:1 and 7.7:1, respectively, when compared to ClHgBr (Table 2). In syntheses involving HgI_2_, to increase its solubility, hot water (80–90 °C) was used to prepare solutions, with the ratios of HgCl_2_ to HgI_2_ and HgBr_2_ to HgI_2_ corresponding to 4:1 and 1:1, respectively.

**Table 1:** Equilibrium Constants for Exchange Reactions Between Mercuric Halides at 25 °C

reaction	log *K*		
	in water^a^	in water^b^	in benzene^c^
HgBr_2_ + HgCl_2_ ⇋2HgBrCl	1.14 ± 0.11	2.0 ± 0.5	2.0 ± 0.5
HgCl_2_ + HgI_2_ ⇋2HgClI	1.35 ± 0.17	1.75 ± 0.20	1.51 ± 0.20
HgBr_2_ + HgI_2_ ⇋2HgBrI	1.07 ± 0.08	1.10 ± 0.20	0.76 ± 0.20

a Low Ionic Strength, Spiro and Hume.^44^

b High Ionic Strength, Marcus.^46^

c Marcus.^46^

**Table 2:** Initial and Calculated Equilibrium Ratios of Simple and Mixed Halides in Reaction Mixtures, Along with Ratios of Ion Signals Measured for Products of Exchange Reactions Between Different HgX_2_ and HgY_2_

X	Y	synthesis temp. (°C)	molar ratio HgX_2_: XHgY: HgY_2_		sample temp. (°C)	signal ratios^b^ HgX_2_: XHgY: HgY_2_	
			initial	at equilib.^a^		SF_6_^–^	HNO_3_·NO_3_^–^
Cl	Br	23	16:0: 1	7.7:1: 0.01	23	4.0:1: 0.07	4.0:1: 0.08
Cl	I	80–90	4:0: 1	1.6:1: 0.03	23	8.0:1: 0.11	N/A^c^: 1:0.15
Br	I	80–90	1.3:0: 1	0.4:1: 0.2	23	N/A: 1:0.25	N/A: 1:0.22
					35	N/A: 1:0.59	N/A: 1:0.72

a Calculated using the equilibrium constants at 25 °C from Spiro and Hume.^44^

b For each chemical, the total signal was calculated by summing up the contributions from all of the isotopic peaks.

c Not available as it was not recorded in the same experiment.

Two variants of the same procedure were used for the synthesis of XHgNO_2_, where X is Cl, Br, or NO_2_, with major differences being in the reagent mixing and product isolation procedures and the amount of water used for the synthesis.^47,48^ In both cases, Hg­(NO_2_)_2_ and ClHgNO_2_ were prepared in a single step, by reacting mercuric chloride with silver nitrite in a 2:1 and 1:1 ratio through reactions 2a and 2b, respectively. BrHgNO_2_ was produced via a two-step procedure, which involved the conversion of mercuric chloride to mercuric nitrite in reaction 2a with silver nitrite, using a 1:2 ratio, followed by the addition of mercuric bromide in a 1:1 ratio to form BrHgNO_2_ (reaction 3). A two-step procedure was used, with HgCl_2_ in the first step, because AgCl is significantly less light-sensitive than AgBr. Additionally, HgCl_2_ is more soluble than HgBr_2_, allowing to produce a more concentrated product by using a smaller volume of mercuric compound solution to compensate for the lower r.t. solubility of AgNO_2_ (0.026 M^49,50^ ).

2a $${HgCl}_{2}+{2AgNO}_{2}\to 2AgCl↓+Hg{\left({NO}_{2}\right)}_{2}$$

2b $${HgCl}_{2}+{AgNO}_{2}\to AgCl↓+{ClHgNO}_{2}$$

3 $$Hg{\left({NO}_{2}\right)}_{2}+{HgBr}_{2}⇋{2BrHgNO}_{2}$$

In the first variant, during synthesis, HgCl_2_ solution was slowly added to AgNO_2_ solution with stirring, and the mixture was left for 2 h to allow the insoluble AgCl to mature and precipitate fully. The clear solution above the precipitate was decanted, vacuum-filtered, and split into two portions. The first portion was evaporated upon heating to obtain crystalline Hg­(NO_2_)_2_ that was used in detection experiments. The second portion was reacted with 1.54 mM HgBr_2_ solution in a 1:1 stoichiometric ratio and then evaporated upon heating. The aqueous solution of HgBrNO_2_ tended to hydrolyze partially upon heating, forming an orange-red precipitate, HgO. Therefore, we also attempted using CH_2_Cl_2_, which is polar and immiscible with water, to extract BrHgNO_2_ from the aqueous solution instead of using evaporative crystallization. After extraction and liquid-layer separation, CH_2_Cl_2_ was allowed to evaporate, leaving behind a colorless solid reaction product that was used for detection.

In the second approach, we followed a modified synthesis procedure reported by Rây^48^ for the preparation of Hg­(NO_2_)_2_. For the synthesis of ClHgNO_2_ and Hg­(NO_2_)_2_, we mixed HgCl_2_ and AgNO_2_ at HgCl_2_:AgNO_2_ molar ratios of 1:1 and 1:2, respectively. The solids were ground in a mortar with a small amount of water to form a paste. The paste was then washed off with a small amount of water into a vial, vigorously mixed and centrifuged to separate AgCl. The supernatant was decanted, filtered, and evaporated using a rotary evaporator with the water bath maintained at 20 °C. To prepare BrHgNO_2_, the dried Hg­(NO_2_)_2_ product was mixed with HgBr_2_ at a 1:1 molar ratio and processed with a minimal amount of water as described above. In all cases, the final product was placed inside a glass container equipped with a vacuum stopcock and dried in a 0.5 Torr vacuum produced by an oilless scroll pump. To minimize oxidation and hydrolysis, these chemicals were stored in evacuated glass containers in the freezer. Additionally, we synthesized ClHgNO_3_ and BrHgNO_3_ by mixing Hg­(NO_3_)_2_ with HgCl_2_ and HgBr_2_, respectively, in a molar ratio of 1:1 in water, followed by crystallization and drying.

During all of the synthesis experiments, standard safety protocols for handling mercury-containing samples were followed, and no unexpected or unusual safety hazards were encountered.

### 2.3 Preparation of Samples for Analysis

Samples for Raman analysis were prepared by placing a small amount of solid material on a glass slide. Spectra were acquired by a DXRxi Raman Microscope (Thermo Scientific), using a 532 nm laser at a power of 1.0–3.0 mW, an acquisition time of 0.5–1.5 s, depending on the sample, and averaging up to 100 scans to achieve the highest possible signal-to-noise ratio. Samples for ID-CIMS analysis were prepared by two different methods. In the first method, several droplets of a product solution were applied to the glass wool placed inside a 1 in. outer diameter Pyrex injector tube and then dried under vacuum, producing glass wool impregnated with the corresponding chemical. In the second method, a crystalline solid was packed in the injector tube between two plugs made of deactivated glass wool or cotton wool; the length of the packed solid column varied between 2 and 10 cm. Commercial mercuric nitrate monohydrate was used as is.

When using the first method with ClHgBr, the impregnated glass wool provided a constant flux of this compound’s vapor released at room temperature over a period of a week. With ClHgI and BrHgI, such a source could be used only for a relatively short time due to the low loading of the glass wool caused by the low solubility of HgI_2_ used in synthesis, producing a low concentration solution. Thus, the second method was used for the mixed halides containing iodine in most detection experiments, providing a larger amount of ClHgI and BrHgI. In several detection experiments with the mixed and simple mercuric nitrites, the sample was held in a glass container equipped with vacuum stopcocks and its headspace was swept into the drift tube by a helium flow at room temperature.

### 2.4 Ion Drift–Chemical Ionization Mass Spectrometer

Simple and mixed mercuric compounds were detected using ID-CIMS,^11,51^ consisting of an ion source, an ion drift tube, and a mass spectrometer, as shown in Figure 1. In the ion source, reagent ions SF_6_^–^, I^–^, and HNO_3_·NO_3_^–^ were produced by sending part-per-million (ppm) levels of SF_6_, CH_3_I, and HNO_3_ in N_2_ (343 standard centimeters per second, sccm) through a negative corona discharge (−800 to −1100 V) at a 2 Torr pressure. The reagent ions entered the ion drift tube, where they reacted with the gaseous analyte, which was generated by sending a ∼ 10 sccm He flow through the mercuric compound-impregnated glass wool or crystalline powder. When using the iodide reagent ion for the detection of mixed and simple nitrites, the headspace of the sample was swept into the drift tube by a flow of helium at room temperature. In several cases, the sample was heated to increase the saturation vapor pressure of the mercuric compound. The drift tube operated under the same pressure as the ion source, and a 10–20 V cm^–1^ electric field was used to constrain ion trajectories and velocities. The corresponding electric-field-to-number-density ratio E/N was 20–29 Td, where 1 Td = 1 Townsend = 10^–17^ V cm^2^. The reagent ions and product ions were detected by a quadrupole mass spectrometer operating at a unit-mass nominal resolution. The unambiguous assignment of the ion composition was performed by comparing experimental and predicted mass spectra, using unique isotope distributions of mercury and halogens.

**Figure 1:**
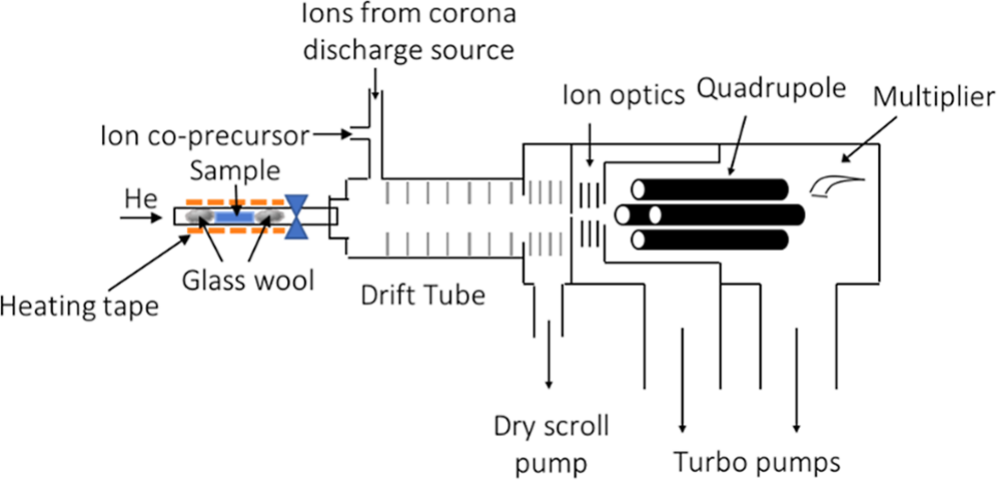
Schematic depiction of the ion drift chemical ionization mass spectrometer with a sample tube loaded with a mercuric analyte. The pressures in the drift tube, the ion optics chamber, and the quadrupole and multiplier chamber are 2.0, 10^–3^, and 10^–6^ Torr, respectively. The ion coprecursor inlet was sealed off in this study.

For SF_6_^–^, the main signal was at 146 amu, accompanied by a small amount of SF_5_^–^ at 127 amu (Figure S1a). For nitrate, the major peak was HNO_3_·NO_3_^–^ at 125 amu, along with minute amounts of NO_3_^–^ and (HNO_3_)_2_·NO_3_^–^ at 62 and 188 amu, respectively (Figure S1b). The ratio between the latter ions depended on the corona discharge voltage, the electric field in the drift tube, and HNO_3_ concentration.^11^ For I^–^, the dominant reagent ion signal was observed at 127 amu, and the corresponding spectra and additional details can be found in our recent study.^52^

### 2.5 Calculation of Ion–molecule Rate Constants

The ADO theory^53–55^ was used to calculate the rate constants of reactions between gas-phase mercuric halides and two of the three reagent ions used in this work

4 $$k_{ADO}=\left(2\pi q/\mu^{1/2}\right)\lfloor \alpha^{1/2}+C\mu_{D}{\left(2/\pi k_{B}T\right)}^{1/2}\rfloor$$

Here, *q* is the ion charge, μ is the reduced mass of the reactants, α and μ_*D*_ are the polarizability and permanent dipole moment of the mercuric halide molecule, *k*_B_ is the Boltzmann constant, *T* is the temperature, and *C* is the dipole locking constant (ranging from 0 to 1) that describes the average orientation of dipole of the neutral molecule in the ionic electric field of incoming reagent ions. The locking constant is a unique function of μ_*D*_/α^1/2^ at constant temperature. The permanent dipole moment and polarizability were taken from quantum chemical calculations of molecules conducted using the Gaussian 09 suite,^56^ similar as in our previous study.^11^ The geometry optimization of all species was executed at the DFT level using the hybrid meta exchange–correlation functional M06–2X, which has been demonstrated to provide reliable structures for Hg molecules.^57,58^ The relativistic effective core potentials were employed to treat the innermost 10, 28, 60 electrons for Br, I, and Hg, respectively. The augmented correlation consistent basis set (aug-cc-pVTZ-pp) was applied to deal with residual electrons in the above atoms and in the Cl atom.^59–61^ We used the ultrafine pruned integration grid and very tight convergence criterion with the root-mean-square force set at 1 × 10^–6^. To ensure all optimized geometries were minima on the potential energy surfaces, the vibrational frequencies were also evaluated. The α for all compounds was an average of the exact and approximate polarizabilities obtained from output files.^62^

The ADO rate constants were used to calculate theoretical detection sensitivity

5 $$S_{t}=R\times k_{ADO}\times t\times f_{iso}\times C_{1ppbv}\left(cps{ppbv}^{-1}\right)$$

where *R* is the reagent ion count rate, *t* is the ion drift time, *f*_iso=_ 0.22–0.30 is the ratio of the relative intensity of the monitored isotope peak to the sum of relative intensities of all isotope peaks in the product ion, and *C*_1ppbv_ = 6.80 × 10^7^ molecules cm^–3^ is the concentration of the analyte gas at a mixing ratio of 1 ppbv at a total pressure of 2.0 Torr and temperature of 298 K. The ion drift time (∼0.8–0.9 ms) was calculated using the reduced ion mobilities of reagent ions together with the electric field and gas flow velocity.

## 3 Results and Discussion

### 3.1 Detection of Mixed Mercuric Halides

In our previous study,^29^ using Raman spectroscopy, we have shown that mixing HgCl_2_ and HgBr_2_ in a 1:1 ratio produced ClHgBr quantitatively as the major product. Figure 2 shows the mass spectrum of ClHgBr from a sample that was prepared using a large excess of HgCl_2_ over HgBr_2_ (16:1) and analyzed by ID-CIMS with SF_6_^–^. A similar mass spectrum obtained using the HNO_3_·NO_3_^–^ reagent ion is shown in Figure S2. The three clusters of peaks with different signal intensities in each cluster belong to three different mercuric compounds. The presence of peak clusters instead of single peaks is due to six naturally abundant isotopes in Hg and two isotopes in Br and Cl. To assign these spectra, we employed an online calculator developed by Scientific Instrument Services.^63^ The relative peak abundances obtained in the experiments are consistent with predicted isotope distributions of HgCl_2_·F^–^, ClHgBr·F^–^, and HgBr_2_·F^–^ (Figure 3) and HgCl_2_·NO_3_^–^, ClHgBr·NO_3_^–^, and HgBr_2_·NO_3_^–^ (Figure S3). These product ions can form if both reagent ions react with HgX_2_ and XHgY via ligand switching.

$$XHgY+{SF}_{6}^{-}\to {XHgY\cdot F}^{-}+{SF}_{5}$$ 6

$${HgX}_{2}+{SF}_{6}^{-}\to {HgX}_{2}{\cdot F}^{-}+{SF}_{5}$$ 7

$$XHgY+{HNO}_{3}{\cdot NO}_{3}^{-}\to {XHgY\cdot NO}_{3}^{-}+{HNO}_{3}$$ 8

$${HgX}_{2}+{HNO}_{3}{\cdot NO}_{3}^{-}\to {HgX}_{2}{\cdot NO}_{3}^{-}+{HNO}_{3}$$ 9

**Figure 2:**
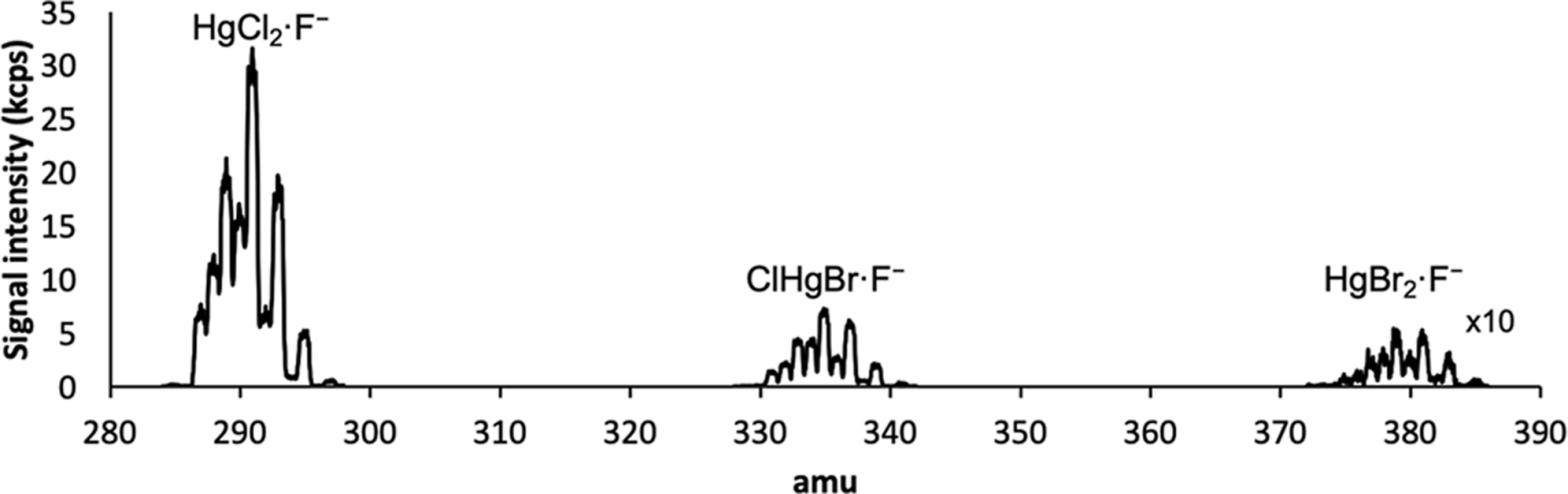
Mass spectra of product ions corresponding to mixed mercuric halide ClHgBr and its precursors—simple halides HgCl_2_ and HgBr_2_. The reagent ion is SF_6_^–^ with peak intensity of 8.3 Mcps, as calculated from the intensity ratio of isotope peaks at 146 and 148 amu. The sample flow rate is 9.6 sccm, and the sample was kept at 23 °C. To improve visualization, the signal corresponding to HgBr_2_F^–^ is magnified by a factor of 10.

**Figure 3:**
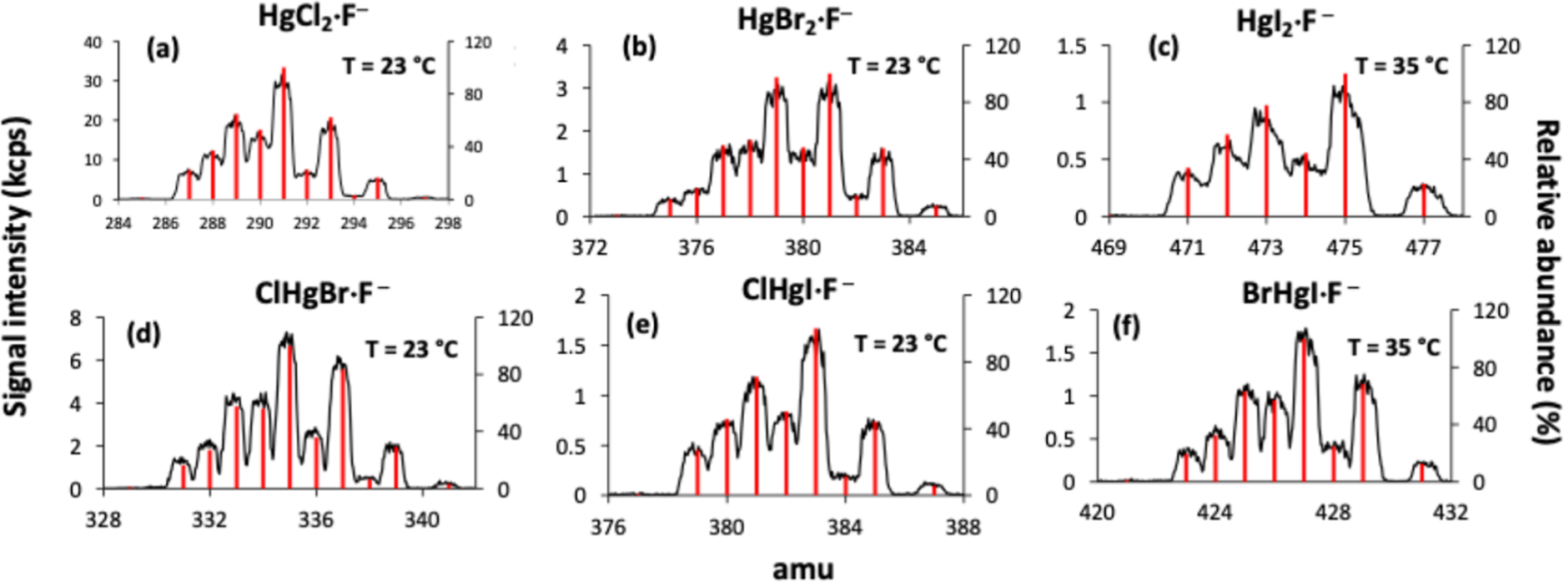
Mass spectra of ions produced through ion–molecule reactions of SF_6_^–^ with simple mercuric halides, HgX_2_ (a–c) and mixed mercuric halides, XHgY (d–f). Calculated mass spectra are shown by red vertical lines. Note that HgI_2_ and BrHgI were heated to 35 °C to increase volatility.

No products of charge transfer from SF_6_^–^ were detected, e.g., HgCl_2_^–^ or ClHgBr^–^, consistent with our previous work^11^ and in agreement with predictions by Dibble et al.^64^ The slow charge-transfer rates in reactions involving SF_6_^–^ have been attributed to a kinetic barrier due to the large structural difference between the anion and neutral forms of SF_6_; only the molecules with electron affinities greater than approximately 2.0 eV show a fast charge transfer in the reaction with SF_6_.^65,66^

Figure 3 shows mass spectra of all the simple and mixed mercuric halides studied in this work, obtained using SF_6_^–^. Similar mass spectra obtained using the HNO_3_·NO_3_^–^ reagent ion are presented in Figure S3. Product ions formed from mercuric compounds containing iodine, i.e., ClHgI, BrHgI, and particularly HgI_2_, show simpler mass spectra with fewer peaks because iodine has only one stable isotope, unlike Cl and Br that have two isotopes. As verified from isotope distributions, all the observed mass spectra correspond to the product ions formed via ligand switching. These results demonstrate that all three XHgY can be readily volatilized from the solid state into the gas phase at room temperature or upon moderate heating while retaining their molecular structures.

### 3.2 Detection of Other Mercuric Compounds

The successful synthesis of mercuric compounds XHgNO_2_, where X is Cl, Br, and NO_2_, was confirmed by Raman spectroscopy, as illustrated in Figure 4.

**Figure 4:**
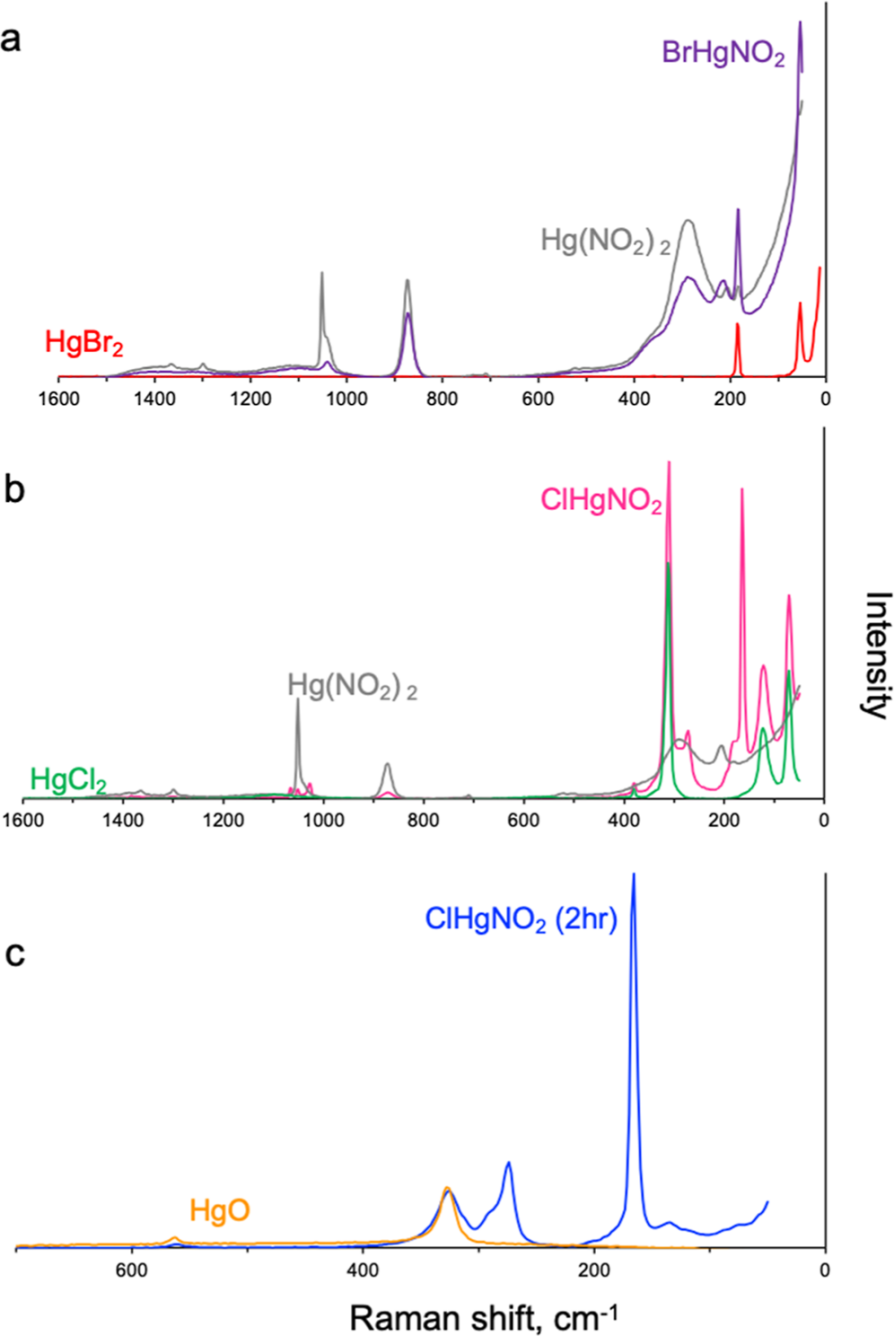
Raman characterization of synthesized mercury nitrite, mercury nitrite halides, and related reference compounds. Raman spectrum of (a) BrHgNO_2_ (purple) compared against Hg­(NO_2_)_2_ (gray) and HgBr_2_ (red), (b) ClHgNO_2_ (pink) compared against Hg­(NO_2_)_2_ (gray) and HgCl_2_ (green), and (c) ClHgNO_2_ after 2 h of exposure to ambient air (blue) compared against HgO (orange).

According to Rây,^48^ Hg­(NO_2_)_2_ has covalent rather than ionic character, and a Raman spectroscopic study by Cram and Davies^67^ has suggested that mercury is coordinated to oxygen in this compound, i.e., has a structure of Hg­(ONO)_2_. Our Raman spectrum of Hg­(NO_2_)_2_ agrees well with that reported by Cram and Davies,^67^ who assigned the vibrations at 880 cm^–1^ and 293 cm^–1^ to δNO_2_ and Hg–O stretching, respectively (Figure 4a). The most common modes of nitrite coordination to metals are monodentate nitrito via oxygen, chelating nitrito via both oxygen atoms, and nitro via nitrogen.^68^ To the best of our knowledge, there is no crystallographic structure available for Hg­(NO_2_)_2_; however, [(18-crown-6)­Hg]­(NO_2_)_2_ has been characterized, and in it, nitrite coordinates to mercury in an asymmetric chelating fashion with Hg–O bond lengths of 2.122 and 2.798 A.^69^ Similar coordination is also the case for Hg­(NO_2_)_4_^2–^, as shown by Hall and Holland.^70^ In vibrational spectra of metal nitrite complexes of the nitro coordination, an out-of-plane wagging mode at relatively high energy (425 to 625 cm^–1^) and M-N stretching frequencies around 300 cm^–1^ to 400 cm^–1^ are expected.^68^ Although the band observed in our Raman spectrum at 384 cm^–1^ falls in an appropriate range for Hg–N stretching, the absence of an additional band for an out-of-plane wagging mode along with structural data on related species suggests that coordination via oxygen is likely, corresponding to Hg­(ONO)_2_.

The spectrum of ClHgNO_2_ shows bands at 872 cm^–1^ and 293 cm^–1^ (Figure 4a), and the spectrum of BrHgNO_2_ shows corresponding bands at 876 cm^–1^ and 293 cm^–1^ (Figure 4b). Both ClHgNO_2_ and BrHgNO_2_ show peaks that are mostly similar to the homoleptic species; however, for ClHgNO_2_, an additional sharp peak was observed at 163.9 cm^–1^, while for BrHgNO_2_, an additional broad peak was observed at 219.9 cm^–1^. All the mercuric nitrite species form red HgO over time with exposure to air, and the Raman spectrum of the decomposition product in Figure 4c shows peaks at 565 cm^–1^ and 330 cm^–1^ that correspond to HgO, as well as 276 cm^–1^ and 166 cm^–1^ that may belong to intermediate decomposition species.

In addition to Raman spectroscopy characterization, we made several observations that clearly distinguished Hg­(NO_2_)_2_ from the mixed mercury nitrite halides ClHgNO_2_ and BrHgNO_2_. The solid Hg­(NO_2_)_2_ product was yellowish, similar to that described by Rây,^48^ whereas ClHgNO_2_ and BrHgNO_2_ were colorless. ClHgNO_2_ and BrHgNO_2_ were highly soluble in methanol, while Hg­(NO_2_)_2_ showed poor solubility in methanol but dissolved readily in water. Hg­(NO_2_)_2_ also appeared to be more stable toward exposure to ambient air than the mixed mercury nitrite halides, although all samples eventually formed reddish crystalline crust. In the case of ClHgNO_2_ and BrHgNO_2_, significant transformation of a thin layer on a glass slide occurred within about an hour. Hg­(NO_2_)_2_ was more hygroscopic than the mixed mercury nitrite halides, remaining sticky even after 2 h under 0.5 Torr vacuum, but could be dried in ambient air when spread in a thin layer on a glass slide. Similar as described by Rây,^48^ Hg­(NO_2_)_2_ produced a distinct nitrous odor and was not stable in air, even in a desiccator, transforming into basic mercuric nitrate over time. Indeed, we detected the presence of significant concentrations of HONO, NO_2_, and HNO_3_ in the headspace of containers holding the three nitrites. The ID-CIMS analysis with SF_6_^–^ and I^–^ as reagent ions showed the following product ions: NO_2_^–^ at 46 amu, HONO·F^–^ at 66 amu, HONO·I^–^ at 174 amu, HNO_3_·F^–^ at 82 amu, and HNO_3_·I^–^ at 190 amu. Based on this evidence, we can envision that nitrites undergo hydrolysis to release HONO and oxidation to form nitrate upon exposure to humid air. The latter reaction appears to be accelerated due to nitrite being coordinated to mercury; the free ionic nitrite in salts such as NaNO_2_ is relatively stable under comparable conditions. After the nitrite ligand departs as NO_3_^–^, the resulting Hg^2+^ deprotonates water, producing HgOH^+^ and eventually HgO. As acidity increases due to deprotonation, some of the nitrate is degassed as HNO_3_.

Although quantum chemical studies suggest that Hg­(NO_2_)_2_,^71^ XHgNO_2_,^30^ and XHgNO_3_ ^57,72^ (X: Cl, Br) exist in the gas phase, our ID-CIMS analysis of headspace vapor above corresponding solids, using I^–^ and SF_6_^–^ reagent ions, showed no product ion signals that could be assigned to any of these compounds. For instance, Figure 5 shows that in the case of BrHgNO_2_, only halides HgCl_2_, HgBr_2_, and HgBrCl were present in the vapor phase above the product. The signals of these detected chemicals were an order of magnitude lower than in the samples of corresponding pure compounds (Figure 3a,b,d). HgCl_2_ and HgBr_2_ were present in residual amounts because they were used in the synthesis, whereas HgBrCl came from their exchange reaction. Neither BrHgNO_2_^–^ nor BrHgNO_2_·F^–^, expected as products of the electron transfer and fluoride transfer pathways, could be observed even upon heating the sample to 50 °C; theoretical spectra of these two ions are shown in red in Figure 5. Since the ion–molecule reaction of gas-phase BrHgNO_2_ with SF_6_^–^ by fluoride transfer is mildly exothermic (ΔH_Reaction_ = -5.70 kcal/mol), according to quantum chemical calculations (Table S1), this chemical should be detectable. Ionization by clustering with iodide is also exothermic by −43.35 kcal/mol (Table S2). Hence, we conclude that BrHgNO_2_ cannot be volatilized from the condensed phase due to its low vapor pressure. Similarly, we were unable to detect any unique mercuric ion products corresponding to ClHgNO_2_ and Hg­(NO_2_)_2_ in samples of those two compounds. The low vapor pressure of mercuric compounds that contain the NO_2_ group indicates stronger intermolecular interactions relative to the compounds containing Cl and Br atoms only, leaving open a possibility of them having a nitro rather than a nitrite structure. Although the nitro form of BrHgNO_2_ is only 3 kcal/mol less thermodynamically stable than its nitrite form,^64^ this difference may be reversed in the condensed phase. By analogy, nitromethane CH_3_NO_2_ has a significantly higher boiling point (101 °C) when compared to methyl nitrite CH_3_ONO that boils at −12 °C. Notably, the boiling point of methyl bromide, CH_3_Br, is 4 °C.

**Figure 5:**
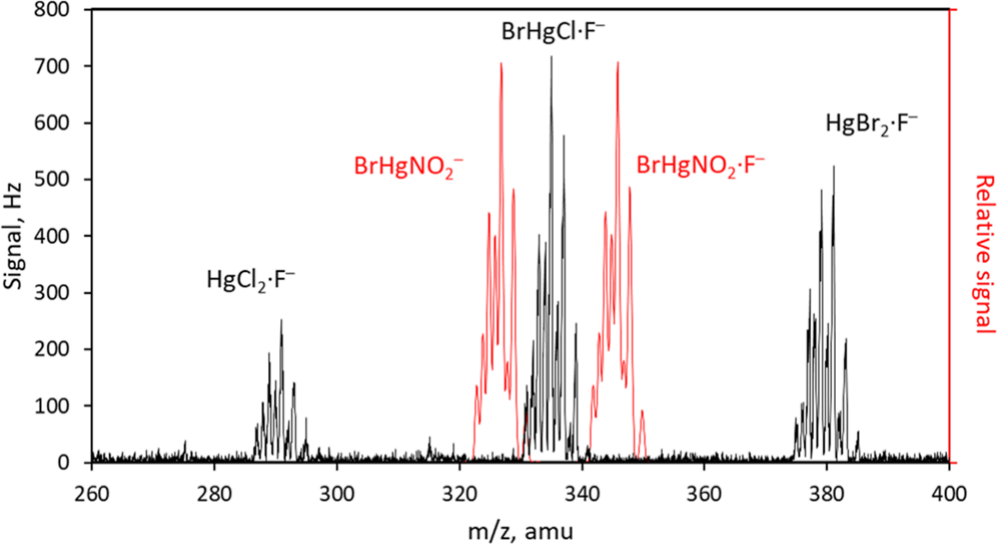
Mass spectra of ions produced through ion–molecule reactions of SF_6_^–^ with chemicals released from the BrHgNO_2_ (black line), along with theoretical spectra of potentially expected ion products (red line).

Using SF_6_^–^ and NO_3_^–^·HNO_3_ as reagent ions, we were unable to detect any unique ion products for samples of ClHgNO_3_, BrHgNO_3_, or Hg­(NO_3_)_2_·H_2_O, whether at room temperature or upon heating to 50 °C. In the case of Hg­(NO_3_)_2_·H_2_O, the sample was heated to 105 °C, resulting in its color changing to brown-red. Apparently, the heating initiated the rapid hydrolysis of mercuric nitrate monohydrate in the reaction with its own water, producing mercuric oxide and nitric acid, where the latter was detected in the mass spectra as HF·NO_3_^–^.

$$Hg{\left({NO}_{3}\right)}_{2}{\cdot H}_{2}O\left(s\right)\overset{\Delta }{\to }HgO\left(s\right)+{2HNO}_{3}\left(g\right)$$ 10

It has been reported that anhydrous mercuric nitrate, which can be prepared by reacting solid HgO with gaseous NO_2_, is somewhat volatile in vacuum.^73^ Apparently, the presence of water in commercial Hg­(NO_3_)_2_·H_2_O makes it an ionic solid (Hg^2+^ and NO_3_^–^), suppressing volatilization. One may surmise that upon heating in high vacuum, dehydration occurs faster than Hg^2+^ hydrolysis, producing a more volatile anhydrous form, as reported by Jones et al.^24^ who observed mass spectra of mercuric nitrate and its fragments during direct probe analysis of Hg­(NO_3_)_2_·H_2_O by electron impact ionization. Such fast dehydration does not occur at ambient pressure, e.g., in a GC column and associated plumbing.^24^

It has been reported that permeation tubes loaded with Hg­(NO_3_)_2_ and HgO emit chemicals that behave like GOM, e.g., can be collected by Hg­(II) traps, such as nylon membranes and KCl-coated denuders,^17^ but the nature of those chemicals is unknown. We surmise that these chemicals could be the traces of volatile mercuric halides present in the samples, or they can be generated through exchange reactions of Hg­(NO_3_)_2_·H_2_O and HgO with trace contaminants present in the carrier gas, e.g., HCl emitted by the fluoropolymer tubing or diffused through the tubing wall from outside air.

### 3.3 Relationship Between Signal and Concentration for Mercuric Halides

In ion–molecule detection reactions occurring via ligand exchange, such as with SF_6_^–^ and HNO_3_·NO_3_^–^ reagent ions, the ion products are fully thermalized and the high-pressure limit ADO value corresponds to the actual rate constant and can be used to calculate detection sensitivity and establish a signal-concentration relationship.^52^ Table 3 shows that the calculated ADO rate constants between different simple and mixed halides fall within 7% of each other. Accordingly, theoretical detection sensitivities estimated for all these chemicals using eq 5 are 13 ± 2 ncps/ppb and 14 ± 2 ncps/ppb for the SF_6_^–^ and HNO_3_·NO_3_^–^ reagent ions, respectively (normalized cps or ncps corresponds to standard reagent ion signal intensity of 1 Mcps).

**Table 3:** Rate Constants of Ion–Molecule Reactions of Mercuric Halides with the SF_6_^–^, HNO_3_·NO_3_^–^, and I^–^ Reagent Ions Calculated by ADO Theory

molecule	polarizability (Bohr^3^)	dipole moment (D)	*k*_ADO_ (10^–10^ cm^3^ molecule^–1^ s^–1^)		
			SF_6_^–^	HNO_3_·NO_3_^–^	I^–^
ClHgBr	85.5	0.53	8.5	9.0	9.0
ClHgI	97.3	1.16	9.8	10.4	10.3
BrHgI	109.8	0.63	9.3	9.9	9.8
HgCl_2_	85.8	0	8.6	9.0	9.0
HgBr_2_	92.4	0	8.5	9.0	8.9
HgI_2_	128.3	0	9.7	10.3	10.2

With nearly identical sensitivities, experimentally measured signals can be used to assess HgX_2_/XHgY/HgY_2_ concentration ratios. These ratios are shown in Table 2, along with molar ratios of these chemicals at equilibrium, as calculated using the equilibrium constants for aqueous solutions. For ClHgBr, reagent ions SF_6_^–^ and HNO_3_·NO_3_^–^ produced similar signal ratios, 4.0:1:0.07 and 4.0:1:0.08, which differed significantly from the 7.7:1:0.01 equilibrium ratio estimated thermodynamically for the sample.^44^ The same applies to ClHgI, which also shows more HgCl_2_ and HgI_2_ in the gas phase above the solid sample relative to the prediction for the aqueous solution (Table 2). We assumed that the molar fractions of mercuric halides in the solid are representative of the molar fractions in solution, but it is possible that the compositions of the solid phase and remaining supernatant solution varied during crystallization, and hence the crystals formed in the beginning and at the end of crystallization had different compositions. Furthermore, gas-phase measurements do not necessarily reflect the average composition of a mixture of those crystals but instead show the most volatile components.

## 4 Conclusions

We demonstrate that mixed mercuric halides ClHgBr, ClHgI, and BrHgI are sufficiently volatile to be detected in the gas phase by chemical ionization mass spectrometry, using SF_6_^–^ and HNO_3_·NO_3_^–^ reagent ions. The ion–molecule reactions occur via F^–^ and NO_3_^–^ ion switching to form unique ion products. Since the ion products are fully thermolized,^52^ the ADO-predicted rate constants can be used to calculate detection sensitivity and establish a signal-concentration relationship. However, mercuric compounds where one or both ligands are not halogens were not detected in the vapor phase, indicating their low volatility or low thermal stability. In the samples composed of simple and mixed mercuric halides, trends between measured signal ratios agree with trends in the theoretical composition ratios calculated using previously reported HgX_2_ + HgY_2_ ⇌ XHgY equilibrium constants. By using a large excess of one monohalide, it is possible to fully deplete the other monohalide and obtain samples composed of one simple halide and a mixed halide. Such samples can be used for the development of detection methods and studying atmospheric mercury chemistry. Solid ClHgNO_2_ and BrHgNO_2_ rapidly oxidize upon exposure to ambient air, and the oxidation is accompanied by their hydrolysis to form red HgO. We conclude that BrHgNO_2_, proposed as a major form of atmospheric GOM, while stable in the gas phase at atmospheric temperatures, is not sufficiently redox-stable to survive multiday preconcentration in condensed phase on sorbents and is insufficiently volatile to be thermally desorbed for analysis.

## Supplementary Material



## Notes

Mass spectra of reagent ions and mercuric halide product ions; computed electronic energies, enthalpies, and Gibbs free energies for neutrals and ions BrHgONO, BrHgONO·F^–^, SF_6_^–^, SF_5_, BrHgONO·I^–^, and I^–^ (PDF)

## §

Howard University

## ∥

University of California–Davis.

## ⊥

University of California–Santa Barbara.

## References

[1] Sprovieri F., Pirrone N., Ebinghaus R., Kock H., Dommergue A. (2010). A review of
worldwide atmospheric mercury measurements. Atmos. Chem. Phys..

[2] Obrist D., Kirk J. L., Zhang L., Sunderland E. M., Jiskra M., Selin N. E. (2018). A review of global environmental
mercury processes in response to human and natural perturbations:
Changes of emissions, climate, and land use. Ambio.

[3] Lin C.-J., Pehkonen S. O. (1999). The chemistry of
atmospheric mercury: a review. Atmos. Environ..

[4] Schroeder W. H., Munthe J. (1998). Atmospheric mercury—an
overview. Atmos. Environ..

[5] Driscoll C. T., Mason R. P., Chan H. M., Jacob D. J., Pirrone N. (2013). Mercury as
a Global Pollutant: Sources, Pathways, and Effects. Environ. Sci. Technol..

[6] Tchounwou P. B., Ayensu W. K., Ninashvili N., Sutton D. (2003). Environmental exposure
to mercury and its toxicopathologic implications for public health. Environmental Toxicology: An International Journal.

[7] Aschner M., Aschner J. L. (1990). Mercury neurotoxicity:
mechanisms of blood-brain barrier
transport. Neurosci. Biobehav. Rev..

[8] Jaffe D. A., Lyman S., Amos H. M., Gustin M. S., Huang J., Selin N. E., Levin L., ter Schure A., Mason R. P., Talbot R. (2014). Progress
on Understanding
Atmospheric Mercury Hampered by Uncertain Measurements. Environ. Sci. Technol..

[9] Gustin M. S., Dunham-Cheatham S. M., Lyman S., Horvat M., Gay D. A., Gačnik J., Gratz L., Kempkes G., Khalizov A., Lin C.-J. (2024). Measurement of Atmospheric Mercury: Current Limitations
and Suggestions for Paths Forward. Environ.
Sci. Technol..

[10] Sommar J. O., Shi X., Tang X., Sun G., Lin C. J., Feng X. (2025). Atmospheric
mercury: recent advances in theoretical, computational, experimental,
observational, and isotopic understanding to decipher its redox transformations
in the upper and lower atmosphere and interactions with Earth surface
reservoirs. Atmos. Chem. Phys..

[11] Khalizov A. F., Guzman F. J., Cooper M., Mao N., Antley J., Bozzelli J. (2020). Direct detection of gas-phase mercuric
chloride by
ion drift - chemical ionization mass spectrometry. Atmos. Environ..

[12] Lyman S. N., Gratz L. E., Dunham-Cheatham S. M., Gustin M. S., Luippold A. (2020). Improvements
to the Accuracy of Atmospheric Oxidized Mercury Measurements. Environ. Sci. Technol..

[13] Gustin M. S., Amos H. M., Huang J., Miller M. B., Heidecorn K. (2015). Measuring
and modeling mercury in the atmosphere: a critical review. Atmos. Chem. Phys..

[14] Stratton W. J., Lindberg S. E., Perry C. J. (2001). Atmospheric mercury
speciation: Laboratory
and field evaluation of a mist chamber method for measuring reactive
gaseous mercury. Environ. Sci. Technol..

[15] Landis M. S., Stevens R. K., Schaedlich F., Prestbo E. M. (2002). Development and
characterization of an annular denuder methodology for the measurement
of divalent inorganic reactive gaseous mercury in ambient air. Environ. Sci. Technol..

[16] Huang J., Gustin M. S. (2015). Uncertainties of Gaseous Oxidized Mercury Measurements
Using KCl-Coated Denuders, Cation-Exchange Membranes, and Nylon Membranes:
Humidity Influences. Environ. Sci. Technol..

[17] Huang J., Miller M. B., Weiss-Penzias P., Gustin M. S. (2013). Comparison of Gaseous
Oxidized Hg Measured by KCl-Coated Denuders, and Nylon and Cation
Exchange Membranes. Environ. Sci. Technol..

[18] Tang Y., Wang S., Li G., Han D., Liu K., Li Z., Wu Q. (2022). Elevated Gaseous Oxidized
Mercury Revealed by a Newly
Developed Speciated Atmospheric Mercury Monitoring System. Environ. Sci. Technol..

[19] Gustin M. S., Dunham-Cheatham S. M., Zhang L. (2019). Comparison of 4 Methods for Measurement
of Reactive, Gaseous Oxidized, and Particulate Bound Mercury. Environ. Sci. Technol..

[20] Jokinen T., Gómez Martín J. C., Feinberg A., Mahajan A. S., Plane J. M. C., Ulises Acuña A., Cuevas C., Dávalos J. Z., Quéléver L. L. J., Laurila T. (2026). Direct
observations of atmospheric oxidized mercury speciation in polar areas. Nat. Commun..

[21] Jokinen T., Sipilä M., Junninen H., Ehn M., Lönn G., Hakala J., Petäjä T., Mauldin R. L., Kulmala M., Worsnop D. R. (2012). Atmospheric sulphuric
acid and neutral cluster measurements using CI-APi-TOF. Atmos. Chem. Phys..

[22] Sexauer
Gustin M., Pierce A. M., Huang J., Miller M. B., Holmes H. A., Loria-Salazar S. M. (2016). Evidence for Different Reactive Hg
Sources and Chemical Compounds at Adjacent Valley and High Elevation
Locations. Environ. Sci. Technol..

[23] Ernest C., Donohoue D., Bauer D., Schure A., Hynes A. (2014). Programmable
Thermal Dissociation of Reactive Gaseous Mercury, a Potential Approach
to Chemical Speciation: Results from a Field Study. Atmosphere.

[24] Jones C. P., Lyman S. N., Jaffe D. A., Allen T., O’Neil T. L. (2016). Detection
and quantification of gas-phase oxidized mercury compounds by GC/MS. Atmos. Meas. Technol..

[25] Deeds D. A., Ghoshdastidar A., Raofie F., Guérette E. ´.-A., Tessier A., Ariya P. A. (2015). Development of a Particle-Trap Preconcentration-Soft
Ionization Mass Spectrometric Technique for the Quantification of
Mercury Halides in Air. Anal. Chem..

[26] Shepler B. C., Peterson K. A. (2003). Mercury monoxide:
A systematic investigation of its
ground electronic state. J. Phys. Chem. A.

[27] Calvert J. G., Lindberg S. E. (2005). Mechanisms of mercury
removal by O-3 and OH in the
atmosphere. Atmos. Environ..

[28] Sjoberg S. (1977). Metal complexes
with mixed ligands. 11. The formation of ternary mononuclear and polynuclear
mercury­(II) complexes in the system mercury­(2+) ion-chloride ion-hydroxide
ion. A potentiometric study in 3.0 M sodium perchlorate, chlorine
media. Acta Chem. Scand., Ser. A.

[29] Mao N., Khalizov A. (2021). Exchange Reactions
Alter Molecular Speciation of Gaseous
Oxidized Mercury. ACS Earth Space Chem..

[30] Jiao Y., Dibble T. S. (2017). First kinetic study
of the atmospherically important
reactions BrHġ + NO_2_ and BrHġ + HOO. Phys. Chem. Chem. Phys..

[31] Lam K. T., Wilhelmsen C. J., Dibble T. S. (2019). BrHgO• + C_2_H_4_ and BrHgO• + HCHO in Atmospheric Oxidation of Mercury:
Determining Rate Constants of Reactions with Prereactive Complexes
and Bifurcation. J. Phys. Chem. A.

[32] Horowitz H. M., Jacob D. J., Zhang Y., Dibble T. S., Slemr F., Amos H. M., Schmidt J. A., Corbitt E. S., Marais E. A., Sunderland E. M. (2017). A new mechanism
for atmospheric mercury redox chemistry:
implications for the global mercury budget. Atmos. Chem. Phys..

[33] Shah V., Jacob D. J., Thackray C. P., Wang X., Sunderland E. M., Dibble T. S., Saiz-Lopez A., Černušák I., Kellö V., Castro P. J. (2021). Improved Mechanistic
Model of the Atmospheric Redox Chemistry of Mercury. Environ. Sci. Technol..

[34] Sturges W. T., Barrie L. A. (1988). Chlorine, Bromine and Iodine in Arctic aerosols. Atmospheric Environment (1967).

[35] Jungwirth P., Tobias D. J. (2006). Specific Ion Effects at the Air/Water
Interface. Chem. Rev..

[36] Antonsson E., Patanen M., Nicolas C., Neville J. J., Benkoula S., Goel A., Miron C. (2015). Complete Bromide Surface Segregation
in Mixed NaCl/NaBr Aerosols Grown from Droplets. Phys. Rev. X.

[37] Knipping E. M., Lakin M. J., Foster K. L., Jungwirth P., Tobias D. J., Gerber R. B., Dabdub D., Finlayson-Pitts B. J. (2000). Experiments
and Simulations of Ion-Enhanced Interfacial Chemistry on Aqueous NaCl
Aerosols. Science.

[38] Beattie I. R., Horder J. R. (1970). Gas-phase Raman
spectra of some dihalides of zinc and
mercury, of ‘GaCl_2_’ and of GaCl_2_Br and GaBr_2_Cl. J. Chem. Soc. Inorg.
Phys. Theor..

[39] Batsanov S., Podberezskaya N., Khripin L. (1965). Mercury salts with mixed anions Communication
3. Synthesis and properties of mixed halides. Russian Chemical Bulletin.

[40] Ahmad R., Ali J., Faisal Q. (2015). An experimental study of formation of the mercury mixed
halides HgClBr and HgBrI and of their purity. Orient. J. Chem..

[41] Rastogi R., Dubey B., Agrawal N. (1975). Mixed halides
of mercury (II). J. Inorg. Nucl. Chem..

[42] Ammlung R., Brill T. (1974). The nature of the mixed
halides of mercury (II). Inorg. Chim. Acta.

[43] Castro P. J., Kellö V., Cernušák I., Dibble T. S. (2022). Together,
Not Separately, OH and O3 Oxidize Hg(0) to Hg­(II) in the Atmosphere. J. Phys. Chem. A.

[44] Spiro T. G., Hume D. N. (1961). The Uncharged Mixed Halides of Mercury
(II). Equilibrium
Constants and Ultraviolet Spectra1. J. Am. Chem.
Soc..

[45] Brodsky A. (1929). Zur Elektrochemie
des Mercuroions. Zeitschrift für Elektrochemie
und angewandte physikalische Chemie.

[46] Marcus Y., Dalziel K., Marcker K., Sillén L. G. (1957). MERCURY
(II) HALIDE MIXED COMPLEXES IN SOLUTION. 3. THE UNCHARGED MIXED COMPLEXES. Acta Chem. Scand..

[47] Ma, S. Dictionary of Chemical Substances; Shaanxi Science and Technology Press, 1999.

[48] Rây P. C. L. I. I. (1904). Mercuric nitrite and its decomposition by heat. J. Chem. Soc., Trans..

[49] Yaws, C. L. Yaws’ Handbook of Properties for Aqueous Systems, 2012; . Knovel Norwich, NY.

[50] Mullin, J. W. Crystallization; Elsevier, 2001.

[51] Fortner E. C., Zhao J., Zhang R. (2004). Development of ion
drift-chemical
ionization mass spectrometry. Analytical chemistry.

[52] Bahramsari M. B., Khalizov A. F. (2026). Detection of Gaseous
Mercuric Halides Using Acetate
and Iodide Chemical Ionization Mass Spectrometry. Anal. Chem..

[53] Su T., Bowers M. T. (1973). Theory of ion-polar
molecule collisions. Comparison
with experimental charge transfer reactions of rare gas ions to geometric
isomers of difluorobenzene and dichloroethylene. J. Chem. Phys..

[54] Su T., Bowers M. T. (1973). Ion-polar molecule collisions: the effect of ion size
on ion-polar molecule rate constants; the parameterization of the
average-dipole-orientation theory. Int. J. Mass
Spectrom. Ion Phys..

[55] Bass L., Su T., Chesnavich W., Bowers M.-T. (1975). Ion-polar molecule collisions. A
modification of the average dipole orientation theory: The cos θ
model. Chem. Phys. Lett..

[56] Gaussian 09, Revision C.01; Gaussian 09: Wallingford, CT, 2009.

[57] Auzmendi-Murua I., Castillo A. l., Bozzelli J. W. (2014). Mercury oxidation via chlorine, bromine,
and iodine under atmospheric conditions: thermochemistry and kinetics. J. Phys. Chem. A.

[58] Jiao Y., Dibble T. S. (2015). Quality Structures, Vibrational Frequencies, and Thermochemistry
of the Products of Reaction of BrHg• with NO2, HO2, ClO, BrO,
and IO. J. Phys. Chem. A.

[59] Dunning T. H. (1989). Gaussian
basis sets for use in correlated molecular calculations. I. The atoms
boron through neon and hydrogen. J. Chem. Phys..

[60] Woon D. E., Dunning T. H. (1993). Gaussian basis sets
for use in correlated molecular
calculations. III. The atoms aluminum through argon. J. Chem. Phys..

[61] Peterson K. A., Puzzarini C. (2005). Systematically convergent basis sets for transition
metals. II. Pseudopotential-based correlation consistent basis sets
for the group 11 (Cu, Ag, Au) and 12 (Zn, Cd, Hg) elements. Theor. Chem. Acc..

[62] Zhao J., Zhang R. Y. (2004). Proton transfer reaction rate constants
between hydronium
ion (H3O­(+)) and volatile organic compounds. Atmos. Environ..

[63] Manura, J. ; Manura, D. Isotope distribution calculator and mass spec plotter by Scientific Instrument Services. 2009. https://www.sisweb.com/mstools/isotope.htm (accessed April 2026).

[64] Dibble T.
S., Zelie M. J., Mao H. (2012). Thermodynamics of reactions of ClHg
and BrHg radicals with atmospherically abundant free radicals. Atmos. Chem. Phys..

[65] Grimsrud E. P., Chowdhury S., Kebarle P. (1985). Electron affinity of
SF6 and perfluoromethylcyclohexane.
The unusual kinetics of electron transfer reactions A–+B =
A+B–, where A = SF6 or perfluorinated cyclo-alkanes. J. Chem. Phys..

[66] Huey L. G., Hanson D. R., Howard C. J. (1995). Reactions of SF6- and I- with Atmospheric
Trace Gases. J. Phys. Chem..

[67] Cram A., Davies M. (1975). LASER-RAMAN STUDY OF SOME MERCURY­(II)
NITRITE COMPOUNDS. J. Inorg. Nucl. Chem..

[68] Hitchman M. A., Rowbottom G. L. (1982). Transition
metal nitrite complexes. Coord. Chem. Rev..

[69] Williams N. J., Hancock R. D., Riebenspies J. H., Fernandes M., de Sousa A. S. (2009). Complexation of Mercury­(I) and Mercury­(II)
by 18-Crown-6:
Hydrothermal Synthesis of the Mercuric Nitrite Complex. Inorg. Chem..

[70] Hall D., Holland R. V. (1969). The crystal structure of K3­[Hg­(NO2)­4]­NO3. Inorg. Chim. Acta.

[71] Li X., Yan M., Dibble T. S., Zhang L. (2022). Reaction mechanism and kinetics of
the important but neglected reaction of Hg with NO2 at low temperature. Chem. Eng. J..

[72] Hewa
Edirappulige D. T., Cheng L., Du H., Song Z., Castro P. J., Zhang Y., Dibble T. S. (2026). Negligible Role
of Nitrate Radical in Directly Initiating Oxidation of Hg(0) to Hg­(II)
in the Atmosphere. ACS Earth Space Chem..

[73] Kozin, L. F. ; Hansen, S. Royal Society Of, C. Mercury Handbook: Chemistry, Applications and Environmental Impact; Royal Society of Chemistry, 2013.

