# Supplementary material for: Detection of Atmospherically Relevant Mixed Mercuric Compounds by Chemical Ionization Mass Spectrometry
Source: ACS Earth Space Chem. 2026 May 19;10(6):1593–602. doi: 10.1021/acsearthspacechem.6c00061 (PMC13288913; doi:10.1021/acsearthspacechem.6c00061)
Supplement: Supplementary file 1 [file sp6c00061_si_001.pdf]

Supporting information for

Detection of atmospherically relevant mixed mercuric compounds by chemical ionization mass spectrometry

Mohammad Borna Bahramsari,[1] Duyen B. Nguyen,[1#] Na Mao,[1$] Joel Duzha,[1&] Michael S. Eberhart,[1] and Alexei F. Khalizov [1,2]*

[1] Department of Chemistry and Environmental Science, New Jersey Institute of Technology, 161 Warren Street, Newark, New Jersey 07102, United States

[2] Department of Chemical and Materials Engineering, New Jersey Institute of Technology, 161 Warren Street, Newark, New Jersey 07102, United States

[#] Now at Howard University

[$] Now at the University of California – Davis

[&] Now at the University of California – Santa Barbara

* Corresponding author (khalizov@njit.edu)

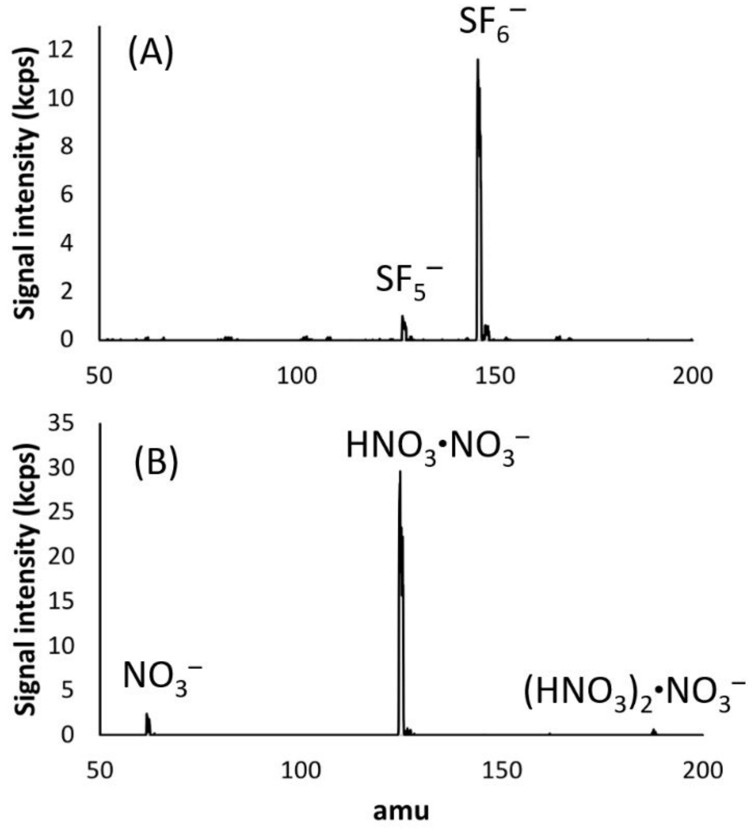

Figure S1: Mass spectra of reagent ions produced via corona discharge from (A) HNO_3 in N_2 and (B) SF_6 in N_2. The signal was detuned to avoid multiplier saturation.

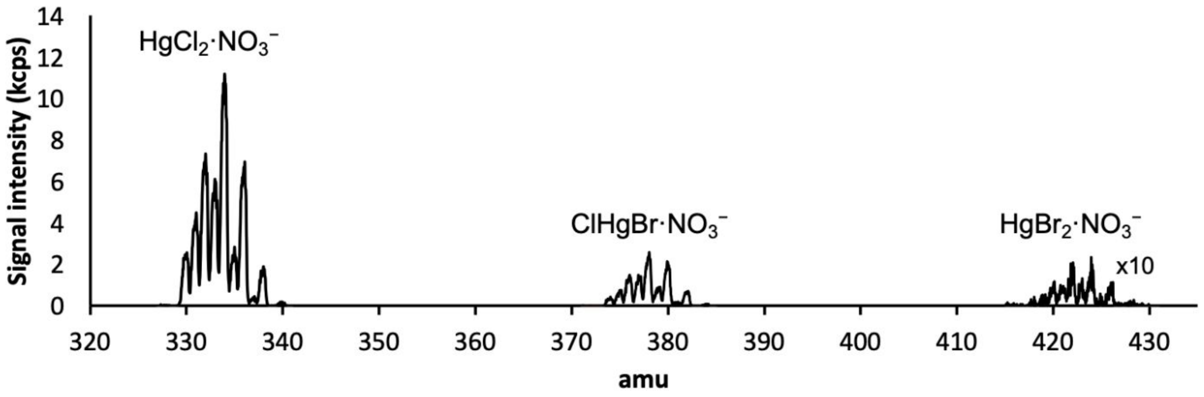

Figure S2: Mass spectra of product ions corresponding to ClHgBr and its precursors, $HgCl_2$ and $HgBr_2$. The reagent ion is $HNO_3 \cdot NO_3^-$ with peak intensity of 14.1 Mcps, as calculated from the intensity ratio of isotope peaks at 125 and 127 amu. The sample flow rate is 9.6 sccm and the sample was kept at 23 °C. To improve visualization, the signal corresponding to $HgBr_2NO_3^-$ is magnified by a factor of 10.

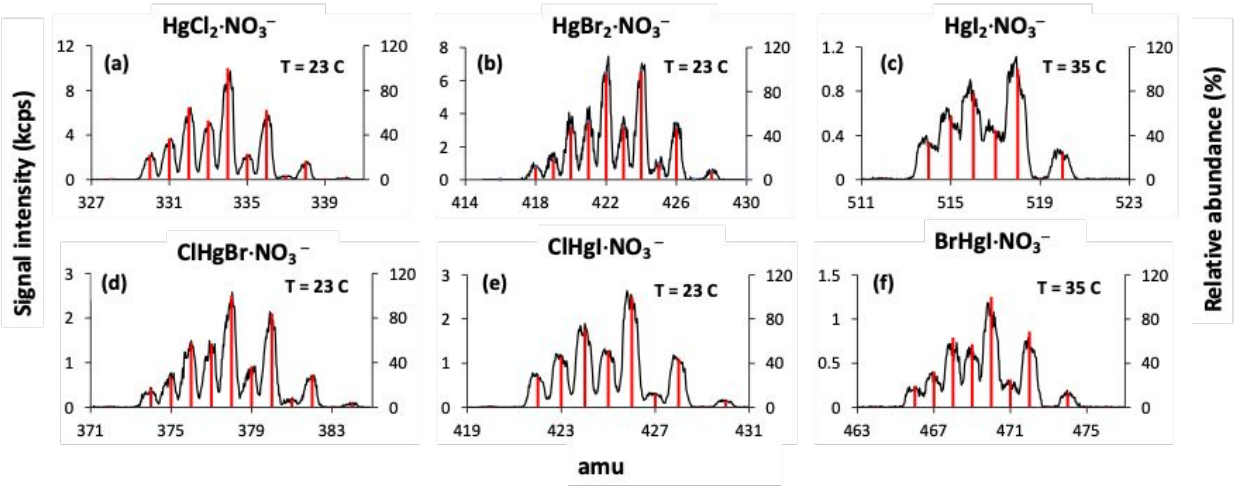

Figure S3: Mass spectra of ions produced through ion-molecule reactions of $HNO_3 \cdot NO_3^-$ with HgX_2 (a, b, c) and XHgY (d, e, f). Calculated mass spectra are shown by red vertical lines.

Table S1: Electronic energies (E), enthalpies (H), and Gibbs free energies (G) calculated for BrHgONO, $BrHgONO \cdot F^-$, SF_6^-, and SF_5. Absolute energies are reported in Hartree, and relative energies (ΔE, ΔH, ΔG) for reaction $BrHgONO + SF_6^- \rightarrow BrHgONO \cdot F^- + SF_5$ are given in kcal mol^{-1}.

**Molecule**	**E**	**H**	**G**	**ΔE**	**ΔH**	**ΔG**
	Hartree			kcal/mole		
BrHgONO	-775.5557	-775.5371	-775.5782	-6.99	-5.70	-5.82
$BrHgONO \cdot F^-$	-875.5138	-875.4923	-875.5401			
SF_6^-	-997.28497	-997.26205	-997.30362			
SF_5	-897.33793	-897.31593	-897.35091			

Table S2: Electronic energies (E), enthalpies (H), and Gibbs free energies (G) calculated for BrHgONO, $BrHgONO{\cdot}I^-$, and I^-. Absolute energies are reported in Hartree, and relative energies (ΔE, ΔH, ΔG) for reaction $BrHgONO + I^- \rightarrow BrHgONO{\cdot}I^-$ are given in kcal mol^{-1}.

**Molecule**	**E**	**H**	**G**	**ΔE**	**ΔH**	**ΔG**
	Hartree			kcal/mole		
BrHgONO	-775.5557	-775.5371	-775.5782	-43.53	-43.35	-37.63
$BrHgONO{\cdot}I^-$	-1071.344	-1071.323	-1071.374			
I^-	-295.7187	-295.7163	-295.7355			
